# Which Way to Choose for the Treatment of Metastatic Prostate Cancer: A Case Report and Literature Review

**DOI:** 10.3389/fonc.2021.659442

**Published:** 2021-04-26

**Authors:** Xiangwei Yang, Donggen Jiang, Yamei Li, Tianzhi Zhang, Duanya Xu, Xianju Chen, Jun Pang

**Affiliations:** ^1^ Department of Urology, Kidney and Urology Center, Pelvic Floor Disorders Center, The Seventh Affiliated Hospital, Sun Yat-Sen University, Shenzhen, China; ^2^ Department of Pathology, The Seventh Affiliated Hospital, Sun Yat-Sen University, Shenzhen, China

**Keywords:** prostate cancer, metastasis, treatment, case report, literature review

## Abstract

**Background:**

Prostate cancer (PCa) is the second most common cancer among males in the world and the majority of patients will eventually progress to the metastatic phase. How to choose an effective way for the treatment of metastatic PCa, especially in the later stage of the disease is still confusing. Herein we reported the case of a patient diagnosed with metastatic PCa and conducted a literature review on this issue.

**Case Presentation:**

A 57-year-old man with metastatic PCa had been managed by Dr. J.P. since April 2012 when the patient was admitted to the Third Affiliated Hospital of Sun Yat-sen University by aggravating frequent urination and dysuria. The prostate-specific antigen (PSA) concentration was 140 ng/ml, and the diagnosis of PCa was confirmed by prostate biopsy, with Gleason score 4 + 5 = 9. Chest CT and bone scan indicated multiple metastases in the lungs and bones. Triptorelin, bicalutamide, zoledronic acid, and docetaxel were then administered, six cycles later, the metastatic tumors in the lungs disappeared and those in the bones lessened significantly, along with a remarkable reduction in PSA level (< 2 ng/ml). Intermittent androgen deprivation was subsequently conducted until August 2018, when the serum PSA level was found to be 250 ng/ml, again docetaxel 75 mg/m^2^ was administered immediately but the patient was intolerant this time. Instead, abiraterone was administered until March 2019 because of intolerable gastrointestinal side-effects and increasing PSA level. In October 2019, the patient came to our center, a modified approach of docetaxel (day 1 40 mg/m^2^ + day 8 35 mg/m^2^) was administered. Luckily, the PSA level decreased rapidly, the bone pain was greatly relieved, and no obvious side effects occurred. However, four cycles later, docetaxel failed to work anymore, the metastatic tumor in the liver progressed. We proposed several regimens as alternatives, but they were soon denied due to the high prices or unavailability or uncertain effect of the drugs. In addition, the patient’s condition deteriorated speedily and can no longer bear any aggressive treatment. Finally, the patient died of multiple organ failure in August 2020.

**Conclusion:**

The experiences of this case provide valuable evidence and reference for the treatment choices of metastatic PCa, in some circumstances modified and advanced regimens may produce unexpected effects.

## Introduction

Prostate cancer (PCa) was first described as a very rare disease by J Adams in 1853 (1). Now, however, PCa is the second most commonly diagnosed cancer and the fifth leading cause of cancer deaths among males, with the estimated occurrence of approximately 1.3 million new cases and 359, 000 deaths worldwide in 2018 (2). Early localized PCa can be effectively treated by radical prostatectomy or radiotherapy while most PCa will eventually progress to metastatic PCa, leading to a median survival time of approximately 3 years for patients (3, 4). Finding a best way of treatment and personalize strategies for metastatic PCa are worthy of consideration. Herein we reported on a 57-year-old man diagnosed with metastatic PCa in 2012, over the next eight years, various therapeutic methods were involved or considered, making the whole treatment process deserves to be shared and further discussed.

## Case Description

**Figure 1:**
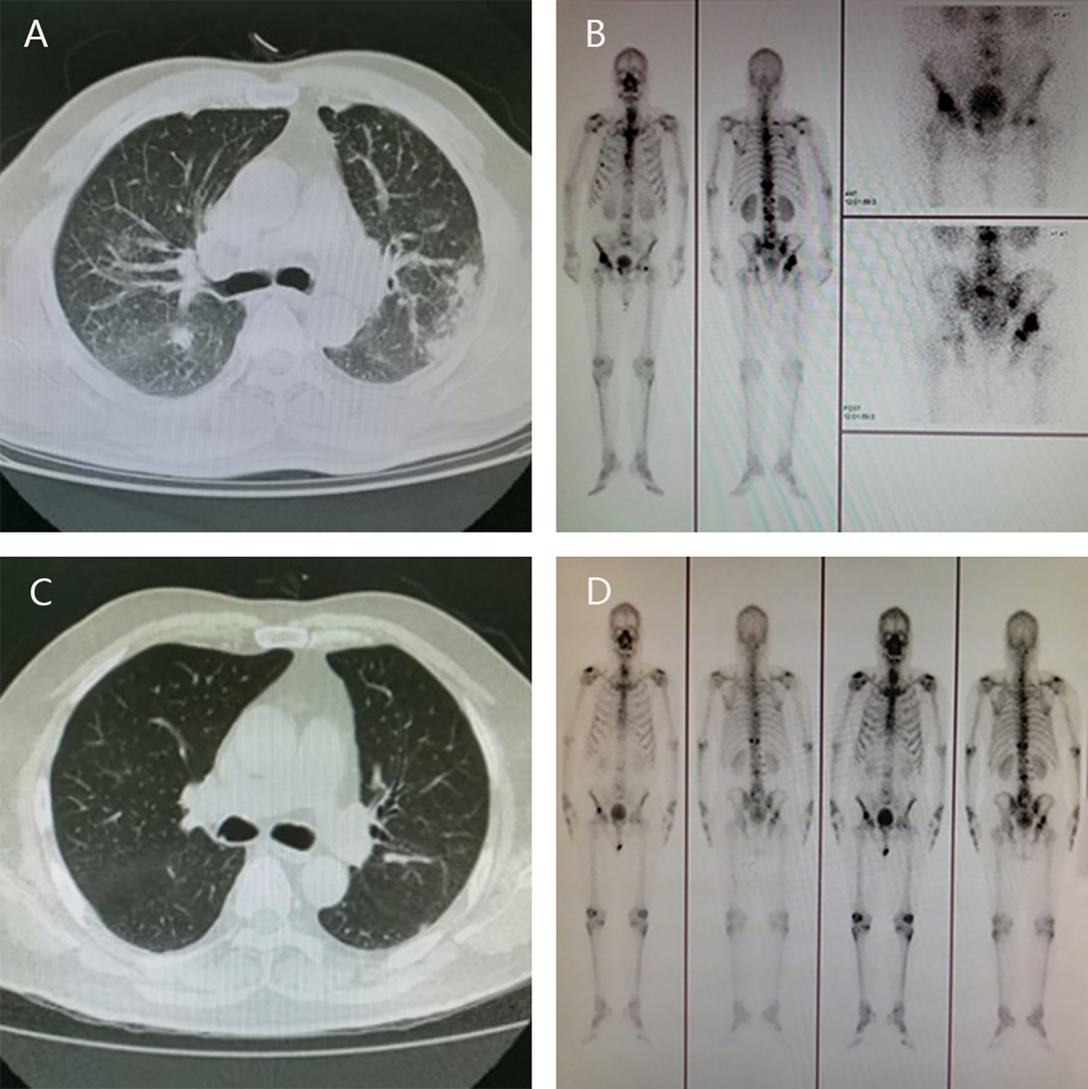
Radiographic change pre- and post-therapy. The results of CT scan showed metastatic tumors in bilateral lungs at diagnosis **(A)** and no visible metastatic tumors in the lungs after androgen deprivation therapy and six cycles of chemotherapy **(C)**; Bone scan showed multiple metastases in the scapulae, ribs, sacroiliac joints, hip joints, thoracic vertebrae, lumbar vertebrae, etc at diagnosis **(B)** and the metastatic tumors in the bones lessened significantly **(D)**.

In April 2012, a 57-year-old man presented with aggravating frequent urination and dysuria was admitted to the Third Affiliated Hospital of Sun Yat-sen University, Dr. J.P. took charge of this patient. Digital rectal examination (DRE) revealed palpable hard nodules and the blood test showed prostate-specific antigen (PSA) concentration was 140 ng/ml. Further ultrasound examination suggested PCa with the right seminal vesicle invasion, chest computed tomography (CT) scan indicated metastatic tumors in bilateral lungs and enlarged lymph nodes in the mediastinum (**Figure 1A**), bone scan demonstrated multiple metastases in the scapulae, ribs, sacroiliac joints, hip joints, thoracic vertebrae, lumbar vertebrae, etc (**Figure 1B**). The diagnosis of PCa was further confirmed by transrectal ultrasound-guided prostate biopsy, with a Gleason score 4 + 5 = 9.

Androgen deprivation therapy (triptorelin) was administered immediately by intramuscular injection, together with anti-androgen (bicalutamide) orally and zoledronic acid intravenously. What’s more, the chemotherapy regimen (docetaxel, 75 mg/m^2^ every 3 weeks) was carried out synchronously, combined with prednisone 5 mg orally twice a day. After six cycles, chest CT and bone scan showed that the metastatic tumors in the lungs were surprisingly disappeared, and the metastatic tumors in the bones lessened significantly (**Figures 1C, D**), along with a remarkable reduction in PSA level (< 2 ng/ml).

Subsequently, namely November 2015, intermittent androgen deprivation (triptorelin combined with bicalutamide) was conducted until 2018, during this period, no regular follow-up was executed for various reasons. In August 2018, the patient was readmitted to hospital due to lumbar compression fractures in an accident fall, his serum PSA level was found to be 75 ng/ml, and rapidly increased to 250 ng/ml 2 months later, implying that the disease had progressed to castration resistance period. A second time he received docetaxel 75 mg/m^2^ immediately but sadly he could not tolerate it, severe fatigue and poor appetite debilitated and troubled him in the extreme, he was much frailer than several years ago. Instead, oral abiraterone was administered, together with prednisone. Fortunately, the PSA level decreased to 15 ng/ml a few months later. However, good times don’t last long, abiraterone was discontinued in March 2019 due to intolerable nausea and vomiting, abdominal pain, and diarrhea. Soon, the PSA level went up to 95 ng/ml, again abiraterone was administered but failed to work, and the PSA level increased to 150 ng/ml, suggesting that the disease was resistant to abiraterone.

**Figure 2:**
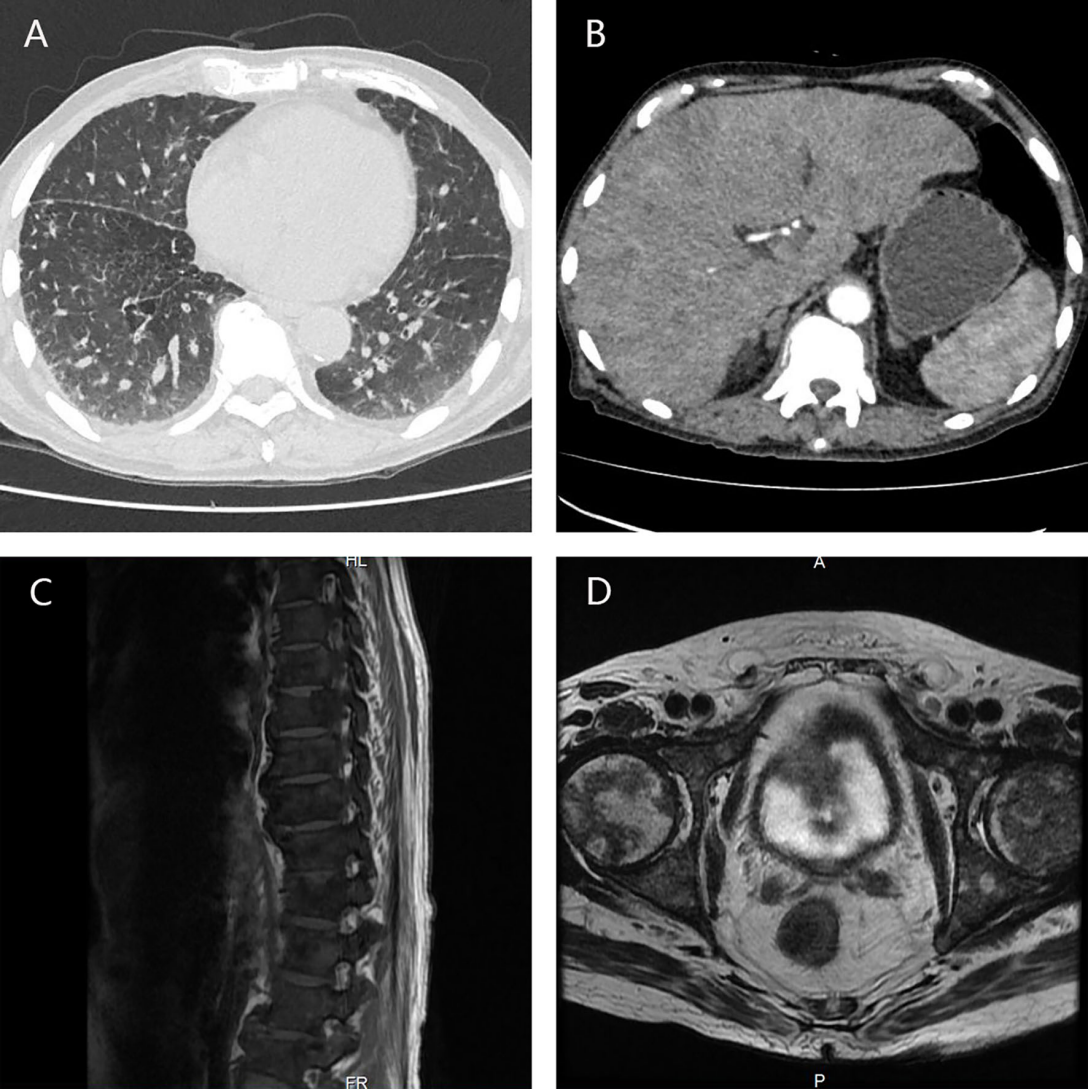
CT/MRI scan showed widespread metastases in the lungs **(A)**, liver, bilateral adrenals **(B)**, thoracic and lumbar vertebrae **(C)**, and pelvis bones **(D)**.

In October 2019, the patient came to our center presenting with poor appetite, general fatigue, and broad bone pain. CT/MRI scan showed widespread metastases in the lungs, liver, bilateral adrenals, thoracic and lumbar vertebrae, and pelvis bones (**Figure 2**), the PSA level was higher than 400 ng/ml. Considering the poor performance status of the patient and the failure experience of abiraterone and standard chemotherapy regimen, we administered docetaxel in a modified approach (day 1 40 mg/m^2^ + day 8 35 mg/m^2^). Luckily, the PSA level decreased rapidly, the bone pain was greatly relieved, and no obvious side effect was observed, the patient regained satisfying appetite and mental status as a consequence.

**Figure 3:**
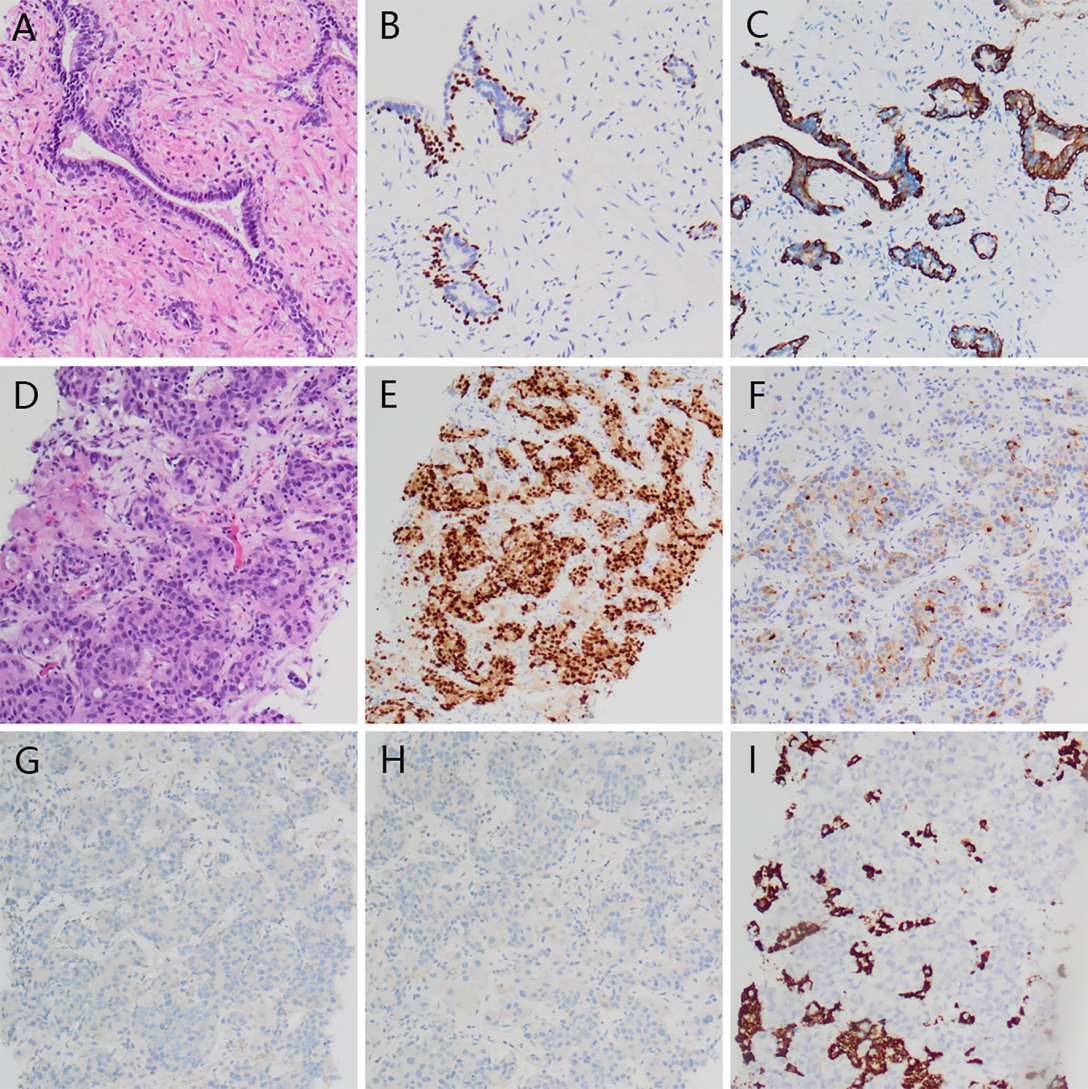
Histopathology of prostate and liver tumor. No visible tumor cell in prostate specimen **(A)**, and expression of P63 **(B)** and 34BE12 **(C)** surrounding the gland. Visible prostate adenocarcinoma in liver tumor **(D)** with expression of AR **(E)**, PSA **(F)** and negative Syn **(G)** and CgA **(H)**, Hepatocyte **(I)**.

**Figure 4:**
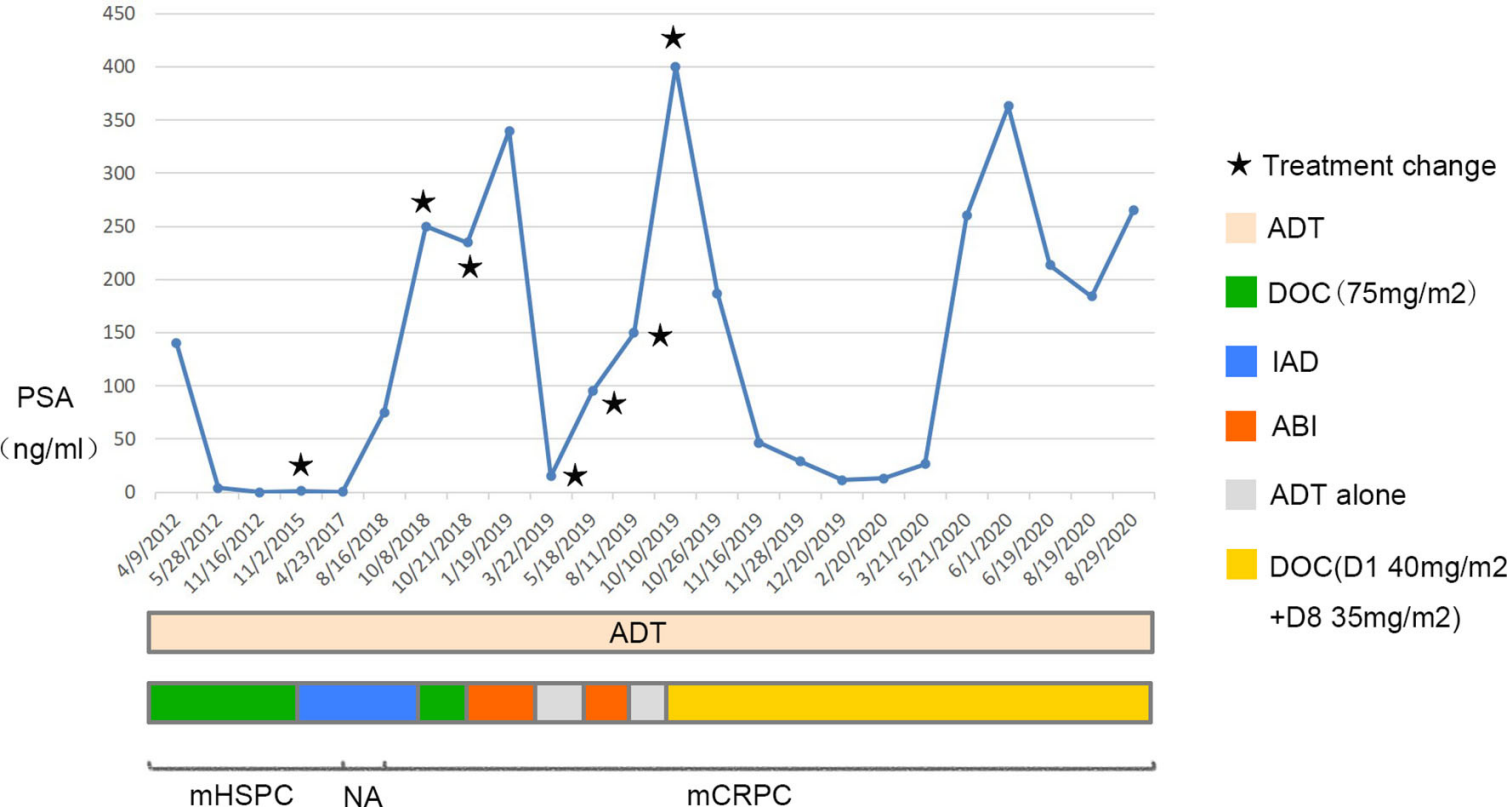
Overall process of disease progression, related treatment and changes of the PSA level. The upper graph shows changes of the PSA level, the treatment course is in the middle and the progression of the disease is shown in the bottom. ADT, androgen deprivation therapy; DOC, docetaxel; IAD, intermittent androgen deprivation; ABI, abiraterone; NA, not available.

Four cycles later, the PSA level decreased to 11.48 ng/ml, the metastatic tumors in the lungs, bones, and adrenals shrank except that in the liver. To determine the pathological type of the prostate cancer and the property of the metastatic tumors in the liver, we performed prostate biopsy and liver biopsy. Consequently, no tumor cell was observed in the specimen of prostate, immunohistochemical stains showed expression of P63 and 34BE12 surrounding the gland (**Figure 3**). In the specimen of liver tumor, prostate adenocarcinoma was observed and the expressions of AR, PSA, P504S, MLH1, MSH2, MSH6, PMS2 were observed in the immunohistochemical stains, with ERG, Syn, CgA, hepatocyte, arginase-1, and PSAP not observed (**Figure 3**). Docetaxel failed to work effectively any more, the PSA level elevated gradually. We took enzalutamide, apalutamide, cabazitaxel, olaparib, and metronomic chemotherapy into consideration as an alternative but soon the proposal was denied because of the high cost or unavailability, uncertain effects of these drugs. In June 2020 and July 2020, we took two more cycles of docetaxel when the patient was back to our center, the PSA level decreased to a certain extent but soon rebounded. At the meanwhile, the patient’s performance status deteriorated speedily, and the total plasma bilirubin level elevated significantly, he could not tolerate any aggressive treatment. Finally, the patient died of multiple organ failure in August 2020. The overall process of disease progression, treatment course and changes of the PSA level were provided below in detail (**Figure 4**).

## Discussion

Improving the outcomes of the patients with PCa is a global health care challenge in recent years (5). In 1941, Charles Huggins and Clarence V. Hodges first introduced endocrine manipulation for metastatic PCa (6). Since then, androgen deprivation therapy (ADT) has been considered as the backbone of treatment for advanced and metastatic PCa (7). Usually, ADT consists of orchiectomy and long-acting luteinizing hormone releasing hormone (LHRH) agonists or antagonists. Comparing the effect of LHRH agonists with orchiectomy, no significant difference was observed in terms of overall survival (OS), but the former was believed to be more acceptable and superior in lowering testosterone levels (8, 9). Even so, orchiectomy remains an effective, inexpensive alternative associated with lower risks of several clinically relevant adverse effects, such as fractures, peripheral arterial disease, venous thromboembolism, etc (10). In a phase III study, LHRH antagonists, a modified decapeptide competitively binding with LHRH receptors, was evaluated and proved to achieve a castrate level much faster than leuprolide in most cases without any flare, and PSA suppression was maintained throughout the whole follow-up period (11). Nevertheless, the definitive superiority of LHRH antagonists over LHRH agonists in OS seems difficult to be concluded (12), and the absence of long-term depot formulations limits the clinical use of antagonists, LHRH agonists are still the mainstream of ADT currently (13).

In terms of timing for ADT, immediate ADT and deferred ADT shared similar cancer specific survival (CSS) while the former was deemed to result in a remarkable increase in OS (14, 15). The latest European Association of Urology (EAU) guidelines recommend immediate ADT as mandatory in symptomatic patients whereas controversy still exists for asymptomatic metastatic patients due to the lack of quality studies (16), higher cost and more frequent treatment-related adverse effects of immediate therapy should be taken into consideration when decisions are made (15). Intermittent or continuous ADT is another concern discussed in several studies, no significant OS inferiority was observed in the intermittent androgen deprivation (IAD) group in contrast to the continuous androgen deprivation (CAD) group (17, 18). But IAD may be more favorable in terms of quality of life (QoL), sexual function, physical activity, cost savings, and treatment-related side-effects (17–19), suggesting that IAD perhaps be a preferred option in some cases, for instance, in the case we presented.

Complete androgen blockade (CAB), a combination of antiandrogen with ADT, has been proved to provide an OS benefit versus ADT monotherapy in a phase III randomized study and several systematic reviews (20–22). While on the other hand, CAB is associated with increased adverse events and reduced quality of life (22). Antiandrogens are often classified as steroidal anti-androgens such as cyproterone acetate (CPA), and non-steroidal anti-androgens (NSAA) such as nilutamide, flutamide, and bicalutamide (16). In a randomized controlled trial (RCT), participants treated with CPA showed similar OS, CSS, and time to progression compared with flutamide, but a lower risk of side effects was observed (23). However, more persuasive studies are currently absent and needed to be further conducted. Comparisons of the efficiency and safety of different NSAA are limited, but bicalutamide was found to show a more favorable safety and tolerability profile than flutamide and nilutamide (24). In our case, a combination of LHRH agonist with bicalutamide may be the most suitable regimen.

Chemotherapy had always been considered unresponsive to PCa until the 1980s. In 1981, Food and Drug Administration (FDA) approved estramustine as the first cytotoxic drug for the treatment of metastatic castration-resistant prostate cancer (mCRPC), followed by mitoxantrone in 1996 (25). Nevertheless, clinical benefits were limited to PSA response, progression-free survival (PFS), and symptoms control, neither estramustine nor mitoxantrone showed OS benefit (26–28). In 2004, docetaxel replaced mitoxantrone as the standard of care based on two well-known phase III studies (TAX 327 and SWOG 9916), for its confirmed benefit on prolonging OS in patients with mCRPC (29, 30). Subsequently, docetaxel was proved to be effective in improving OS in patients with metastatic hormone-sensitive prostate cancer (mHSPC), especially those with high-volume metastatic disease, according to three phase III studies (CHAARTED, STAMPEDE and GETUG-AFU 15) and a systematic review and meta-analysis involved these three trials (31–34). As a consequence, ADT combined with docetaxel is strongly recommended by EAU guidelines as the first-line treatment for those who are initially diagnosed with metastatic PCa and fit for docetaxel (16). On the other hand, neutropenia, fatigue, nausea, and vomiting are common among patients receiving docetaxel (29), in our case, fatigue and poor appetite are the main manifestations in the first time of docetaxel rechallenge. Concerning those who are too frail to tolerate 75 mg/m^2^ docetaxel, what Kellokumpu-Lehtinen P L did may provide an alternative that deserves to be referred to. In his dose-adjusted group, a similar oncological outcome was obtained while fewer adverse events were reported (35). In our case, we successfully proved the efficacy of docetaxel in the treatment of mHSPC (before guidelines), mCRPC, and the feasibility of modified chemotherapy regimen in frail patients, we also validated the benefit of docetaxel rechallenge in patients with mCRPC relapsing after an initial good response to docetaxel, which are consistent with previous studies (36, 37). In the later stages of the disease, docetaxel resistance occurred. The mechanisms of docetaxel resistance have not been explicitly illuminated, possible mechanisms include overexpression of P-glycoprotein, activation of androgen receptor, mutation of β-tubulin, aberrant angiogenesis, etc (38, 39). Therefore, biomarkers test may predict docetaxel response ahead of PSA change. Ploussard et al. proved the patients with the expression of βIII-tubulin had a significant shorter median OS than those with negative βIII-tubulin (40), other promising biomarkers include interleukin-6, macrophage inhibitory cytokine 1 and so on, but further studies are needed to confirm the clinical value (41, 42). The methods to overcome docetaxel resistance have also been discussed, alternative drugs such as cabazitaxel or enzalutamide are also good choices, nanotechnology mediated docetaxel delivery may also produce a surprising outcome (38, 39).

In patients with mHSPC, abiraterone, enzalutamide, and apalutamide are another first-line treatment choices according to EAU guidelines (16), all of which have shown significant improvements in OS and PFS than standard ADT in previous studies (43–45). In terms of abiraterone and docetaxel, existing evidence shows that abiraterone is comparative or even superior to docetaxel on oncological outcomes (46–50), and the former might be associated with higher QoL and less treatment-related toxicity (49, 50). Even though, as we reported, abiraterone may also produce severe side-effects, such as vomiting, abdominal pain, and diarrhea (50). Further contrastive data among different available first-line regimens are currently insufficient, several factors should be taken into account when making a treatment decision, including disease volume, comorbidities, patient preference, toxicity profile, availability, and cost, etc (51).

Similarly, in patient with mCRPC, abiraterone, and enzalutamide were proved to significantly prolong OS and PFS in several randomized double-blind phase 3 studies and therefore were listed on the first-line treatment regimens (16, 52, 53). Apalutamide showed a significant metastasis-free survival (MFS) benefit among men with nonmetastatic castration-resistant prostate cancer (54), and good safety and efficacy in patients with mCRPC according to several small-size studies (55, 56), while further randomized phase 3 studies are needed to draw a more persuasive conclusion. Sipuleucel-T is another comparative first-line choice, which has shown its efficacy in prolonging OS among men with mCRPC, accompanied with tolerable adverse events (16, 57). Usually, abiraterone and enzalutamide are used prior to docetacel, and abiraterone -to-enzalutamide sequence was more favorable in terms of PFS (58). Interestingly, though, abiraterone and enzalutamide were confirmed to significantly prolong the survival of men with mCRPC after docetaxel (59, 60). Detection of androgen-receptor splice variant 7 (AR-V7) was proved to be associated with resistance to abiraterone and enzalutamide (61), while the negative conversion of AR-V7 following docetaxel has been reported, the discovery may explain the benefit of abiraterone and enzalutamide following docetaxel, and consequently abiraterone rechallenge may function as usual (62). On the other hand, enzalutamide showed a modest response rate in castration-resistant prostate cancer patients progressing after the use of abiraterone, similar clinical outcomes were observed in the application of abiraterone after enzalutamide failure, which implied cross-resistance was not inevitable (63, 64).

Cabazitaxel, a second-generation taxane developed to overcome docetaxel resistance, was approved in 2010 for the treatment of patients with mCRPC who had previously received docetaxel-based regimens (25), for its superiority over mitoxantrone in terms of clinical responses and OS (65). However, in patients with chemotherapy-naïve mCRPC, cabazitaxel did not show superiority for OS compared with docetaxel (66), therefore, docetaxel remains the first-line chemotherapeutic option for this population (16). Regarding the adverse events, cabazitaxel and docetaxel demonstrated different toxicity profiles, cabazitaxel may offer additional flexibility in patients with neuropathy, edema, or other conditions that may preferentially be exacerbated by docetaxel (66). In addition, cabazitaxel 20 mg/m^2^ was deemed to be as effective as 25 mg/m^2^, while less toxicity was observed, which suggested a lower dose should be preferred to reduce adverse events (66, 67). In frail elderly patients, metronomic chemotherapy, which is based on more frequent and low-dose drug administrations, such as daily oral vinorelbine and cyclophosphamide, provides an interesting alternative (68, 69), yet much larger, controlled, and prospective clinical trials are needed to figure out the optimal regimens (70). In patients with DNA-damage repair mutations in genes such as BRCA1, BRCA2, and ATM, Olaparib, a PARP inhibitor, led to a high response rate (71, 72), considering the potential similar mechanisms between olaparib and platinum (73), platinum-based chemotherapy may also be sensitive to this population (71), genetic test may play a valuable guiding role. When bone metastases were confirmed, radium-223 may provide benefit in OS, prolong the time to first skeletal event and improve pain scores and QoL (74). Recently, PD-1 inhibitor pembrolizumab showed antitumor activity and good disease control ability with acceptable safety in patients with docetaxel-refractory mCRPC, regardless of PD-L1 status, which is an encouraging innovation (75).

Bone metastasis and skeletal-related events (SREs) were proved to be associated with poorer prognosis among PCa patients, especially when they occurred synchronously (76, 77). Zoledronic acid was the first agent shown to decrease SREs according to a randomized placebo-controlled trial and therefore was approved by the FDA in 2002, with the recommended regimen of 4 mg every 3 weeks (25, 78). In 2011, a novel agent named denosumab, a fully human monoclonal antibody against receptor activator of nuclear factor kappa-B ligand (RANKL) was confirmed better than zoledronic acid for the prevention of SREs (79). Interestingly, both zoledronic acid and denosumab were associated with increased bone mineral density among men receiving ADT for nonmetastatic PCa (80, 81), and denosumab was showed to offer benefit of delaying bone metastasis *via* changing the bone microenvironment in a large randomized study (82). Nevertheless, hypocalcemia was more frequent with denosumab versus zoledronic acid, all serum calcium deficiency should be corrected before and during treatment with bone protective agents (83).

## Conclusion

Some limitations exist in our treatment course, including the absence of genetic or biomarker test for drug selection, and the deficiency of regular follow-up data. While on the other hand, individual or practical factors could not be ignored, personalized strategies are needed, together with systematic regimens. On the premise of ADT, the efficacy, toxicity, cost, availability of treatment regimens, and patients’ preference should be taken into consideration. For some peculiar patients, modified and advanced regimens may produce unexpected effects.

## Data Availability Statement

The original contributions presented in the study are included in the article/**Supplementary Material**. Further inquiries can be directed to the corresponding author.

## Ethics Statement

Written informed consent was obtained from the individual(s) for the publication of any potentially identifiable images or data included in this article.

## Author Contributions

XY and DJ: manuscript writing and data collection. YL, TZ, DX, and XC: data collection. JP: project development and data collection. All authors contributed to the article and approved the submitted version.

## Funding

The present study was funded by the Sanming Project of Medicine in Shenzhen (SZSM202011011), and Research start-up fund of part-time PI, SAHSUSY (ZSQYJZPI202003).

## Conflict of Interest

The authors declare that the research was conducted in the absence of any commercial or financial relationships that could be construed as a potential conflict of interest.
