# Supplementary figures and images for: Which Way to Choose for the Treatment of Metastatic Prostate Cancer: A Case Report and Literature Review
Source: Front Oncol. 2021 Apr 26;11:659442. doi: 10.3389/fonc.2021.659442 (PMC8107685; doi:10.3389/fonc.2021.659442)

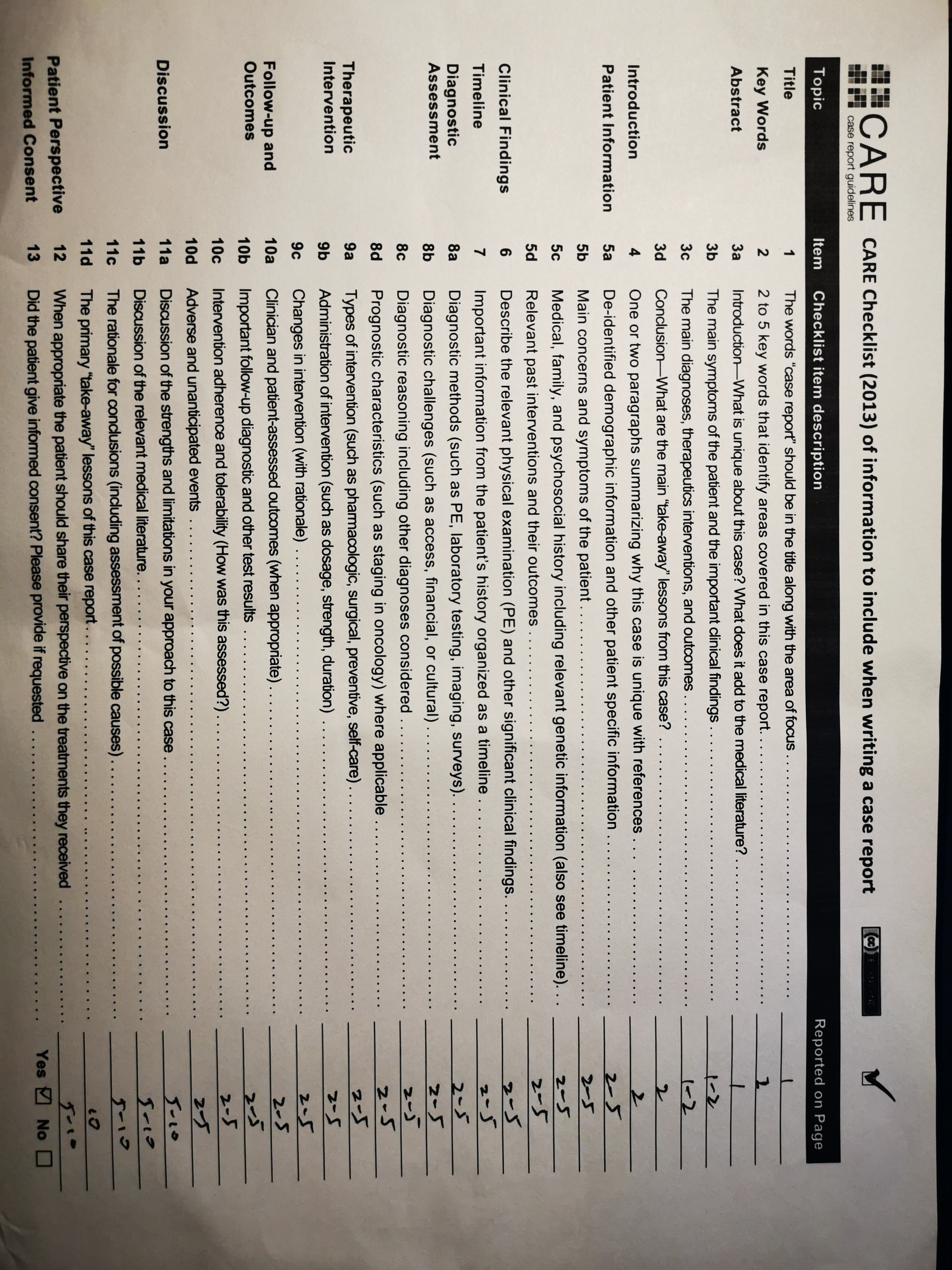

Supplement: Supplementary file 1 [file Image_1.jpeg]
